# Predicting Functional Recovery in Chronic Stroke Rehabilitation Using Event-Related Desynchronization-Synchronization during Robot-Assisted Movement

**DOI:** 10.1155/2016/7051340

**Published:** 2016-01-17

**Authors:** Marco Caimmi, Elisa Visani, Fabio Digiacomo, Alessandro Scano, Andrea Chiavenna, Cristina Gramigna, Lorenzo Molinari Tosatti, Silvana Franceschetti, Franco Molteni, Ferruccio Panzica

**Affiliations:** ^1^Institute of Industrial Technology and Automation (ITIA), National Research Council (CNR), Via Bassini 15, 20133 Milan, Italy; ^2^University of Brescia, Via Branze 38, 25123 Brescia, Italy; ^3^Department of Neurophysiology, Fondazione IRCCS Istituto Neurologico Carlo Besta, Via Celoria 11, 20133 Milan, Italy; ^4^Villa Beretta Rehabilitation Center, Via Nazario Sauro 17, 23845 Costa Masnaga, Italy

## Abstract

Although rehabilitation robotics seems to be a promising therapy in the rehabilitation of the upper limb in stroke patients, consensus is still lacking on its additive effects. Therefore, there is a need for determining the possible success of robotic interventions on selected patients, which in turn determine the necessity for new investigating instruments supporting the treatment decision-making process and customization. The objective of the work presented in this preliminary study was to verify that fully robot assistance would not affect the physiological oscillatory cortical activity related to a functional movement in healthy subjects. Further, the clinical results following the robotic treatment of a chronic stroke patient, who positively reacted to the robotic intervention, were analyzed and discussed. First results show that there is no difference in EEG activation pattern between assisted and no-assisted movement in healthy subjects. Even more importantly, the patient's pretreatment EEG activation pattern in no-assisted movement was completely altered, while it recovered to a quasi-physiological one in robot-assisted movement. The functional improvement following treatment was large. Using pretreatment EEG recording during robot-assisted movement might be a valid approach to assess the potential ability of the patient for recovering.

## 1. Introduction

In the last decades, robot-assisted rehabilitation has been used to promote motor ability in stroke patients. Although different studies show promising results in the treatment of both subacute [1, 2] as well as chronic [3, 4] stroke patients, the additive effects of robot-assisted rehabilitation with respect to traditional therapy still have to be demonstrated. It is widely accepted that intensive repetitive task-oriented robotic rehabilitation is an effective intervention [5] but, at present, the mechanisms leading to impairment reduction following the robotic training are still unclear [3]. In addition, it is unknown whenever the reduction in impairment leads actually to improved quantity and quality of upper-limb use in activities of daily living. Finally, there is still no consensus neither on which robot and kind of approach should be used given a selected patient nor on the duration of the robotic intervention [6]. There are various robots and approaches for upper-limb neurorehabilitation but, due to the lack of knowledge of the mechanisms leading to improvement and the complexity and the variety of the clinical pictures of stroke patients, there are no generally recognized guidelines on how to select the robotic intervention and how to customize it on the patient's residual functional abilities. Electroencephalography (EEG), which has been in use for more than 80 years, is a low cost, noninvasive, and versatile technique to assess cortical functional reorganization. EEG is easily applicable and might be used during robotic intervention to evaluate the effects of the motion assistance on the activity of the interconnected brain regions. In fact, the analysis of EEG recorded during a motor performance provides information about the changes in the oscillatory cortical activity related to movement with a good temporal resolution. A well-established and widely applied technique to explore the modulation of EEG rhythms due to the movement is the Event-Related Desynchronization/Synchronization (ERD/ERS) analysis that allows quantifying the movement related power change of the EEG oscillatory activity in alpha and beta bands over the premotor and primary sensorimotor areas [7]. In healthy subjects, amplitude attenuation of specific frequency components (ERD) in alpha and beta bands precedes and lasts during voluntary movement and reflects cortical activation concurring with movement planning and execution. At the end of the movement, a rebound of activity (ERS) in the beta band replaces ERD indicating the local inhibition of the motor cortex. Better comprehension of the EEG changes before, during, and after assisted-robotic training might help selecting the proper robot interventions along with defining and customizing protocols in stroke patients' rehabilitation.

The basic idea behind this study was that the considered robotic intervention provides strong afferent stimuli that are important for promoting neural plasticity. In the literature, this type of robotic intervention is not valued because it is considered to be passive since the robot follows a predefined motion law independently of the forces exerted by the subject; instead an* assist as need* approach is preferred [8]. However, the authors of the current work considered that the robotic movements might induce cortical activation even when they are fully assisted. The research hypothesis was tested on a group of healthy subjects by analyzing the effects of this kind of robot assistance on the cortical activation. Namely, a comparison was done between the active voluntary movement and the robot-assisted one by visually analyzing the related ERD alpha and beta maps. Secondly, the hypothesis was tested on a chronic stroke patient. Again, the effects of the robotic intervention on the motor cortex activation were evaluated by visually comparing the EEG data relative to the active movement and the assisted one. The patient underwent the robotic intervention and clinical evaluations were performed after 1-month treatment, at the end of the treatment, and at 1-year follow-up. The clinical outcome data were matched with pretreatment EEG recordings and retrospectively analyzed. Unfortunately, it was not possible to perform the EEG acquisitions after treatment and in the follow-up.

The aim of the present work was to verify the research hypothesis that, in healthy subjects, a functional movement performed with total robot assistance is characterized by the same brain oscillatory activity as the no-assisted movement. Clinical results following the robotic treatment of chronic stoke patients, who showed cortical reorganization during the robotic intervention, are analyzed and discussed.

The work is divided into two parts:

1. The first stage focused on the group of healthy subjects, where EEG data during a functional movement performed actively and with robot assistance were recorded and further analyzed.
2. The second stage focused on the chronic stroke patient, where the clinical results gained thanks to the robotic intervention were retrospectively matched with pretreatment EEG data to evaluate the potentiality of this investigation for being used in the definition process of stroke patients' treatment programs.

## 2. Material and Methods

### 2.1. Participants

Eight healthy subjects (neurologically and orthopedically intact) and one stroke patient with left hemiparesis were included in the study.

The group of healthy subjects included 5 females and 3 males; the average age was 32 ± 12 years. All subjects were right-hand dominant (Edinburgh Handedness Inventory Test [9]).

The patient was a 65-year-old woman, right-hand dominant, with left hemiparesis due to an ischemic right lenticular infarction involving the internal capsule. At the beginning of the treatment, she was already in the chronic phase of the disease, namely, at 7 months from the acute stroke event.

Written informed consent was obtained from each subject before inclusion in the study. The study was reviewed and approved by the local Ethics Committee at Como Valduce Hospital and was conducted in compliance with the Declaration of Helsinki.

### 2.2. Equipment

**Figure 1:**
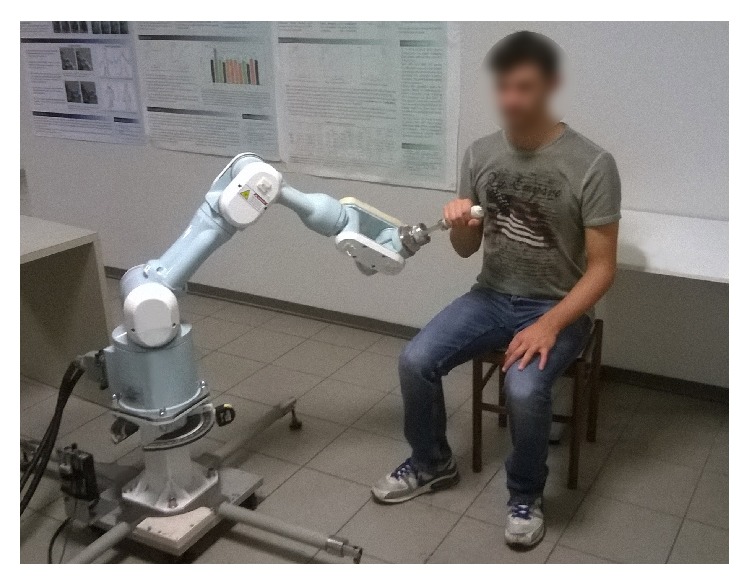
The Mitsubishi Pa10-7 robot platform.

- An end-effector based industrial robot (Pa10-7, Mitsubishi, Japan) customized for rehabilitation purposes (see Figure 1) allows for the execution of functional movements performed at physiological velocity [10].
- A 3D-motion capture system (6 TVc, Smart-D, BTS, Italy) was used to track the active (no-assisted) performed movement in order to define the motion law to be used to program the robot for assistance.
- An EEG-EMG acquisition system (SynAmps^2^ model 8050, Compumedics Neuroscan, Charlotte, NC, USA) was used.

### 2.3. EEG Assessment

#### 2.3.1. Motor Task and Protocol

**Figure 2:**
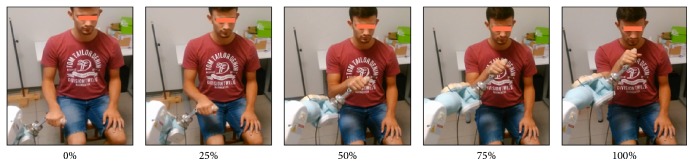
The robot-assisted Hand-to-Mouth Movement.

A paradigmatic upper-limb functional gesture, namely, the Hand-to-Mouth Movement (HtMM, see Figure 2) [11], was chosen for EEG evaluation. The HtMM-motion laws (path and velocity) of the active (no-assisted) movements performed by each subject were acquired using the 3D-motion capture system and the procedure described in Caimmi et al. 2012 [10]. In order not to get robot-tracking errors (which appear when the robot is unable to follow the given motion law), the maximum velocity was limited and the whole velocity profile was rescaled. Therefore, the robot-assisted movement durations can vary among subjects due to the different anthropometric measures. In the case of the patient, the movement was generated using the motion law acquired during HtMM performed using the unaffected upper limb. The path was then mirrored with respect to the patient's sagittal plane to be used for the robotic assistance of the affected limb.

The EEG assessment protocol consisted in a single session (unfortunately, it was not possible to perform patient's posttreatment and follow-up EEG acquisitions). During the robot-assisted trials, subjects were asked to actively participate. Holding the robot handle, they had to try to slightly anticipate the movement rigidly imposed by the robot or rather they were asked to behave like they were performing the movement autonomously, without robot assistance [10]. The robot assisted the movement with a physiological (bell-shaped) velocity profile.

During the evaluation session, the following 3 trials were carried out:

- Right hand active no-assisted movement.
- Left hand active no-assisted movement.
- Robot-assisted movement (the right hand in the case of the healthy subjects and the left one in the case of the stroke patient).

At the beginning of the acquisition, 5 minutes of resting state condition was acquired for each subject. For each trial, at least 50 movements were acquired with a random pause between the end of a movement and the onset of the following one of 10 ± 2 s. The choice of using a random pause was taken to avoid premovement cortical activation phenomena. In the robot-assisted movement, the trigger was a signal automatically sent by the robot controller to the EEG acquisition system at beginning and end of movement. In the case of the free performed movements, an operator cued the subject to start moving and, simultaneously, sent the begin and end movement triggers using a computer keyboard.

#### 2.3.2. Acquisition

EEG signals were recorded using a cap providing 64 electrodes positioned according to the International 10/10 System; EMG activity was simultaneously recorded from pairs of Ag/AgCl surface electrodes placed bilaterally 2-3 cm apart over the biceps and the brachioradialis muscles. The EEG and EMG data were acquired using a Neuroscan system at a sampling frequency of 512 Hz (band-pass filters: 1–200 Hz). The ground electrode was placed between Fpz and Fz and the reference electrode between Cz and Cpz.

#### 2.3.3. Data Analysis

Data analysis was conducted by means of software developed in house using the MATLAB language (R2012a, Mathworks Inc., Natick, MA, USA). Artifacts in the EEG recordings (i.e., eye movements and scalp muscle contraction) were removed using an Independent Component Analysis (ICA) [12]. Subsequently, in order to achieve reference-free and spatially sharpened EEG data, the surface Laplacian estimate was applied [13].

Movement offsets were determined using the triggers and, when necessary, by manually tagging the beginning of each burst of EMG activity. EEG data were epoched between −3 s and 7 s with respect to movement onset. For each participant, the most reactive alpha and beta band frequencies were determined, in the 8–13 and 13–30 Hz frequencies ranges, respectively, by means of an iterative procedure (individual reactive frequency, IRF). For the alpha band, the most reactive frequency was chosen maximizing the ERD negative peak. For the beta band, the most reactive frequency was chosen maximizing the difference between the ERD negative peak and the ERS positive peak. Each epoch was digitally band-pass filtered in the IRF −1 Hz to IRF + 1 Hz band by means of a zero-phase 512-point finite impulse-response filter. The filtered EEG signals were then squared, averaged over all epochs, and downsampled with one data-point every 200 ms. The relative ERD/ERS values were expressed as percent power change, calculated with respect to the mean power in the −2.5 s to −1.5 s premovement reference period. In both the individual and group analyses, the statistical significance of the differences between the mean power observed during the reference period and that measured during the subsequent 200 ms intervals was expressed as a probability value using Wilcoxon's signed rank test. The power changes were considered significant when the *p* value was less than 0.05.

A topographic map showing the significant changes in ERD/ERS for the alpha and beta bands was computed for each subject along with the mean map of the healthy group.

A statistical comparison between patient map and mean map of the healthy subjects was performed [14].

### 2.4. Patient's Robotic Treatment

The stroke patients underwent a robot-assisted therapy based on the execution of two functional movements:

1. The HtMM, the one previously evaluated through EEG recording (video 1 in Supplementary Material available online at http://dx.doi.org/10.1155/2016/7051340).
2. The Reaching Movement (RM) against gravity which was considered by the patient's referent physician to be promising to promote shoulder flexion [10] (video 2).

The single session protocol consisted of 20 minutes of assisted HtMM followed by 20 minutes of assisted RM against gravity. Velocities were rigidly imposed; that is, the robot handle followed the predefined path and motion law independently of the forces applied by the patient. In rehabilitation, this kind of intervention is commonly defined as* Continuous Passive Mobilization* (CPM) because the patient does not have to actively participate. In order to avoid this, the patient was explicitly asked to participate by trying to follow (slightly anticipate) the moving handle. The operator, a specialized physiotherapist, could monitor on video the forces of interaction between the patient and the robot and, if necessary, could encourage the patient to try to participate more. In order not to make the exercise too fatiguing (it is worth recalling that both movements were against gravity), the patient was asked to change the level of engagement every 5 movements by alternately relaxing during movement and actively participating. Further, the patient had to actively orientate the robot handle, which was provided by a turning joint, toward the mouth (see Figure 2). The patient had 3 sessions of intervention a week (on Monday, Wednesday, and Friday). The patient underwent 1-month treatment (12 sessions) and, afterwards, given the positive results and the availability from both the patient and the clinical structure side to continue the treatment, the robotic intervention was further prolonged for another 3 months (30 sessions). Therefore, the complete treatment consisted of a total of 42 training sessions performed in 4 months.

### 2.5. Patient's Clinical Evaluation

Clinical evaluations were performed before treatment (T0), after 1 month of treatment (T1), at end of treatment (i.e., after 4-month treatment, T2), and at 1-year follow-up (T3).

The clinical evaluation was based on the following scales:

- The upper-limb section (A–D) of the Fugl-Meyer Assessment (FMA) [15].
- The Manual Muscle Test (MMT), also known as Medical Research Council (MRC) scale for muscle strength [16].

FMA is used to define the global functional impairment of the patient. Muscle strength was evaluated through the MRC.

## 3. Results

### 3.1. EEG

#### 3.1.1. Healthy Subjects

**Figure 3:**
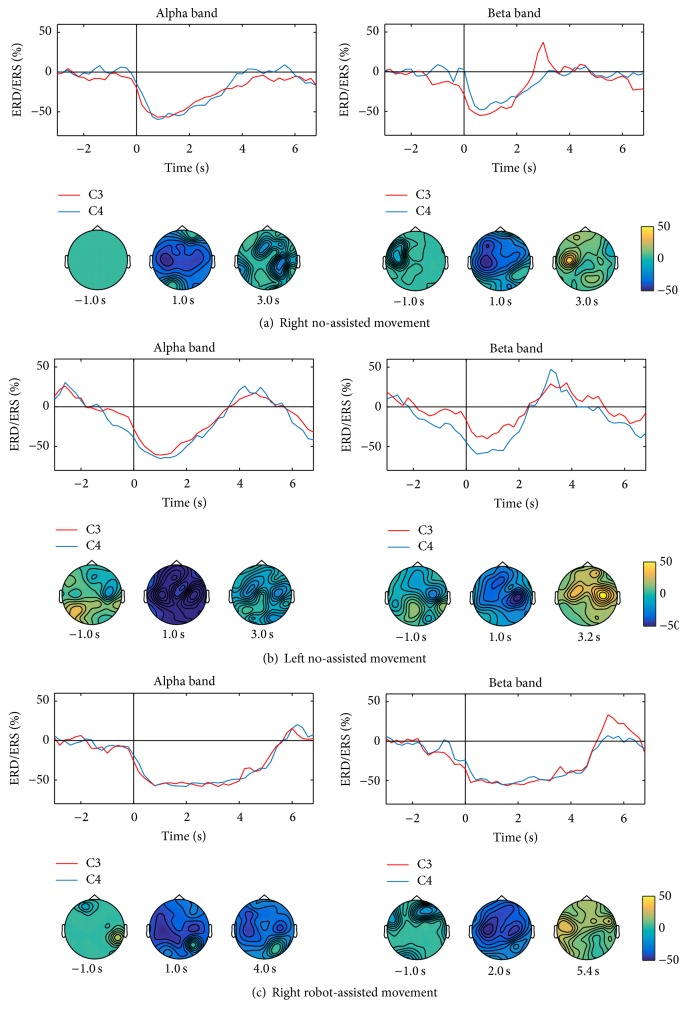
Mean alpha and beta ERD/ERS color maps in control subjects group during right hand active no-assisted movement (a), left hand active no-assisted movement (b), and right hand robot-assisted movement (c). ERD/ERS values reported are expressed as percent power change.

All subjects performed well the motor tasks A, B, and C so that in mean, respectively, 51 ± 8, 53 ± 5, and 51 ± 6 movements matched the selection criteria and were therefore included in the analysis. The mean durations of the movement were 3.00 ± 0.94 s, 2.86 ± 0.73 s, and 5.05 ± 0.68 s for the A, B, and C tasks, respectively. The mean reactive frequencies were 10.3 ± 0.95 Hz (A), 11 ± 1.49 Hz (B), and 11 ± 1.2 Hz (C) in alpha band and 20.2 ± 2.25 Hz (A), 20.3 ± 2.75 Hz (B), and 19.4 ± 3.31 Hz (C) in the beta one. During voluntary active movements (Figures 3(a) and 3(b)), in both alpha and beta bands, the desynchronization started at the movement onset on the contralateral central area, which reached maximal values. In the alpha band, ERD spread toward contralateral frontocentral and ipsilateral central areas and returned at baseline level at the movement offset. Differently, in the beta band, the desynchronization was restrained on the contralateral surface area and replaced by a synchronization peak at the end of the movement. During robot-assisted movement (Figure 3(c)), the desynchronization patterns in the alpha and beta bands started at the onset of the movement bilaterally on the central areas lasting until movement offset. In the beta band, at the movement offset, the desynchronization was replaced by synchronization on the contralateral central area (A: 2.89 ± 0.77 s; B: 2.78 ± 0.61 s; C: 5.22 ± 0.54 s). The maximal values of desynchronization and synchronization in the alpha and beta bands were similar among the three tasks (Table 1).

#### 3.1.2. Patient

**Figure 4:**
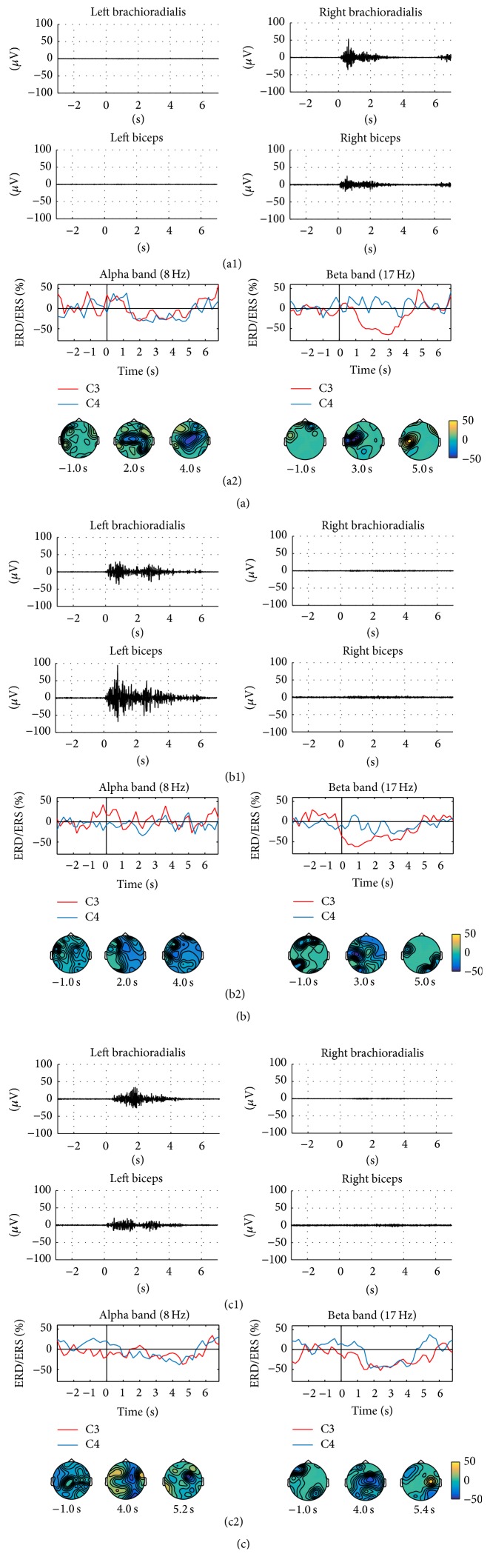
ERD/ERS analysis on the patient. ((a1), (b1), and (c1)) Mean of EMG signals; ((a2), (b2), and (c2)) Alpha and beta ERD/ERS time courses in contralateral and ipsilateral central areas (upper panels) and ERD/ERS color maps during right (unaffected) hand active no-assisted movement (a), left (affected) hand active no-assisted movement (b), and left (affected) hand robot-assisted movement (c). ERD/ERS values reported are expressed as percent power change.

The patient performed all the tasks; 47, 48, and 53 movements were well done and therefore analyzed for the A, B, and C tasks, respectively. The duration of the unaffected hand no-assisted movement duration was comparable with those of the healthy subjects (task A 2.95 ± 0.45 s) and the one of the affected hand no-assisted movement was longer than that of the healthy subject average duration (task B 3.9 ± 0.64 s), while, finally, the one of the affected hand robot-assisted movement was shorter (task C 4.3 ± 0.29 s) (Figures 4(a1), 4(b1), and 4(c1)). Regarding task C, the movement was faster than the healthy subjects' average movement because the patient was a small lady. The most reactive frequencies were 8 and 17 Hz, for the alpha and beta band, respectively. During the active tasks performed with the unaffected hand, alpha and beta ERD/ERS patterns similar to those observed in the control subjects were detected (Figure 4(a), Supplementary Figure  1(a)). Conversely, during the active task performed with the affected hand, the ERD/ERS pattern was barely recognizable in terms of temporal and topographical patterns (Figure 4(b)) and a stable desynchronization pattern could not be identified for both studied bands. Even the postmovement synchronization in the beta band could not be detected. During the robot-assisted movement with the affected hand, a reduced desynchronization pattern in the alpha band was detected on the contralateral central area; in the beta band, the desynchronization involved bilaterally the central areas, whereas the postmovement synchronization was present on the contralateral central area (Figure 4(c)).

### 3.2. Intervention Results

The clinical results of the evaluations at T0, T1, T2, and T3 are reported in Table 2. At T0, before treatment, the patient demonstrated functional impairment at shoulder and elbow (FMA section A = 17/36), wrist (FMA section B = 4/10), and hand (FMA section C = 4/14). The clinical evaluation after 1 month of treatment (T1) showed motor function improvement both at proximal and at distal levels (FMA section A = +6, FMA section B = +1, and FMA section C = +2). Further improvement was recovered during the 3-month extra treatment, again both at proximal and at distal levels (FMA section A = +7, FMA section B = +2, and FMA section C = +3). The clinical evaluation at 1-year follow-up (T3) not only showed retention but also highlighted an extra functional improvement, again, at both proximal and distal levels with exclusion of the wrist (FMA section A = +5 and FMA section C = +3). In summary, the total motor function improvement at T3 measured through the FMA scale was +18/36 points at the proximal joints, +3/10 points at the wrist, and +8/14 points at the hand. The final function measured at T3 was therefore as follows: FMA section A = 35/36 (shoulder and elbow), FMA section B = 7/10 (wrist), and FMA section C = 12/14 (hand).

With regard to the Manual Muscle Test, results at T0 showed slight hyposthenia at the shoulder and elbow joints (MMT = 4/5) and asthenia for finger extension (MMT = 1/5, slight muscle contraction, and absence of movement). Results were confirmed at T1 as no difference in score was found. At the end of treatment (T2), full muscle strength recovery was reached at both shoulder and elbow (MMT = 5/5); by contrast no improvement had appeared in the fingers strength. At 1-year follow-up (T3), the patient was finally able to fully extend the fingers against gravity (MMT = 3/5).

## 4. Discussion

It is widely accepted in neurorehabilitation robotics that to promote function in stroke patients active participation of the subject to movement is needed. In the common view, a robot should provide “*as needed assistance*” or “*minimal assistance*” [17] in order to permit the exploration of the effort-error relationship that stimulates motor relearning [8, 18, 19]. Concurrently, there is evidence in the literature that robotic interventions based on movements against gravity successfully reduce shoulder-elbow impairment in stroke [20]. Unfortunately, the* assist-as-need* principle is often of little applicability in training against gravity, especially in the case of low functioning patients with high strength and coordination impairments. In these cases, when the patient is not able to control actively the robot, “*passive mobilization,*” based on a rigidly imposed trajectory (path and motion law), is the only remaining option. In this framework, the authors wanted to verify the hypothesis they made according to which in healthy subjects a functional movement performed with total robot assistance (and classically considered to be* passive*) is actually accompanied by cortical activation like in the case of the actively (no-assisted) performed movement.

With regard to the healthy subjects, during the HtMM, the movement selected for EEG investigation, cortical activation was apparent even during the robot-assisted movement. Like in the actively performed movement, the ERD/ERS analysis showed bilateral activation over the central sensorimotor areas with predominant contralateral activation in both the alpha and beta rhythms. These data are in line with the few published EEG studies applying the ERD approach during passive motion. A bilateral decrease in the beta rhythm magnitude during passive moment was found also in other two recent studies [21, 22]. Other studies demonstrated that passive movements are characterized by same beta ERD/ERS activity topography as active movements but lacks the premovement ERD [23, 24]. In both studies, the authors concluded that afferent proprioceptive inputs (from joint, tendon, and muscle receptors) might also play a role in the beta ERD observed during voluntary movements. In the present work, premovement ERD components were not present even in the active HtMM because the patient was cued to move by the operator who issued a start some seconds after the end of the previous movement. The break between two movements was, on purpose, of different duration to avoid premovement cortical activity. The EEG data of the present work showed that both the alpha and the beta ERD/ERS activity topographies were similar in the active and robot-assisted movement. It could be questioned whether the subjects were specifically asked to actively participate during the robot-assisted movement but, actually, another study based on passive movement imposed by the robot obtained very similar results [22, 25]. Anyway, results of the present work showed that the robot-assisted (functional) movement in question may not be considered being passive, even when fully assisted. It further suggests that the same somatosensory areas are involved in the motor control of the active as well as the robot-assisted movement. This is the main finding of the work, a first step for validating the idea that robot fully assisted mobilization might provide afferent stimuli even in neurological patients, stimuli that are important to induce brain activity and promote neural plasticity.

With regard to the investigation conducted on the patient in the case of the active HtMM performed with the nonaffected limb, a physiological ERD/ERS pattern was observed in both the alpha and beta bands over the somatosensory central areas, contralateral and ipsilateral to the movement. Conversely, no organized cortical activity was spotted out during the active HtMM performed with the affected upper-limb. Interestingly, when the affected upper-limb HtMM was performed with full robot assistance, an EEG activation pattern was again recognizable. This finding is worth consideration because it seems to suggest that the robot assistance might help cortical reorganization. To the authors' best knowledge, neurological studies based on EEG acquisition during robot fully assisted motion in patients are few, and in only one study on a chronic stroke patient the beta map showed stronger bilateral ERD during the assisted movement as compared with healthy controls [26]. Differently to the results of the current study, organized cortical activity was present also in the active movement since beta mapping showed bilateral ERD although with predominance on the contralateral side (over C3). Whilst one study reported more power of the rhythmic activity within the alpha band during active movement than during the passive one [27]. Establishing the timing and the reasons of the ERD appearance during robot-assisted movement while being absent in the corresponding active movement should be further investigated.

Clinical results following the robotic treatment showed a large improvement in function and strength during the whole treatment. The clinical relevance of the improvement may be evaluated through comparison of the gained function with the Minimum Clinically Important Difference (MCID) [28] of the FMA scale, which specifically is 9 points. It is worth noting that the MCID of the FMA scale was estimated for subacute patients, where spontaneous recovery is ongoing. This makes the 10, 22, and 30 points of FMA improvement obtained at T1, T2, and T3, respectively, even more important. The chronic phase of stroke is characterized by the absence of the spontaneous recovery and functional improvement is gained through exercise. Conversely, decrease in function may happen due to the* learned nonuse phenomenon*. The current results showed further improvements after the end of the treatment (8 FMA points from T2 and T3) because the patient was using the affected upper limb more and more during* Activities of Daily Living* (ADLs) due to the gained functionality. In fact, at T0, the patient presented moderate functionality at shoulder and elbow (FMA = 17/36) but no functionality at the hand (FMA = 4/14, just some finger flexion but no extension at all). At T0, the affected upper limb could not therefore still be used for performance of ADLs. Improved hand (and wrist) function along with regained strength and motor control ability at shoulder and elbow levels cued a virtuous circle where the more use of the affected limb in ADLs induced further functional improvement and so on.

Within the research of this study, a patient with proximal upper-limb moderate impairment and no hand functionality was evaluated through EEG recording. The patient was tested during active and robot-assisted performance of a particular functional movement, namely, the HtMM. Interestingly, the patient reacted positively to the robot assistance showing, at T0, some bilateral cortical activity in the alpha and beta bands which was not recognizable in the actively performed movement. Even more interestingly, the patient showed a functional improvement following treatment beyond any possible positive prevision considering the chronic phase of the disease. A similar case was reported in a study on a patient showing at 1 year after the stroke event some organized cortical activity during* passive* robot-assisted mobilization [26]. In this case, after EEG evaluation, the patient underwent 3-month home intervention based exercises for improving somatic sensation, hand dexterity, and arm function and use. After treatment, the patient showed improved upper-limb somatic sensation and hand dexterity, function, and use along with associated specific changes in the modulation of alpha and beta event-related synchronization/desynchronization. The authors of that work concluded their assessment procedure based on the combination of EEG and robot-assisted upper-limb devices could provide new insights into the dynamics of cortical reorganization promoted by rehabilitation in stroke patients. The results presented within the current research seem consistently to confirm their hypothesis.

## 5. Conclusions

The work presented in this study was performed to verify that fully robot assistance would not affect the physiological oscillatory cortical activity related to a functional movement in healthy subjects. EEG data demonstrated the intervention being effective in producing cortical activity. The alpha and beta bands mappings overlapped the ones during the actively performed movement. This result might show the potentiality of the intervention in stimulating neural plasticity. Cortical activity during robot-assisted movement has shown even in a chronic stroke patient who demonstrated very large motor function recovery after treatment. These are promising results although, at present, being based on 1 patient only, it is not clear whether pretreatment* ERS* in the alpha and beta band might “*simply*” indicate a patient's potential ability for recovering or, more specifically, the particular robotic intervention to be promising in promoting motor recovery in this patient.

This work presents some limitations. The HtMM only was investigated through EEG recording although the patient underwent a treatment based on RM against gravity also. Unfortunately, an investigating protocol based on two movements would have been too heavy for the patient considering the long preparation procedure using 64 electrodes and the number of movement performed in each trial. The possibility of using a reduced number of electrodes (i.e., 19 channels placed according to 10-20 international systems) will be considered for future works. However, EEG data were not actually used in this work for demonstrating the validity of the treatment but to evaluate the patient's potentialities for recovering. Further investigations, based on a different study design, are needed to verify the possibility of using EEG to tailor the (robotic) treatment program to a patient's needs. A further limitation is that no posttreatment EEG data were presented. Unfortunately, it was not possible to organize further EEG acquisitions. The authors acknowledge this is an important lack clearly. Posttreatment EEG recordings were needed to verify how the cortical reorganization happened and, specifically, if there were correlations between the alpha and beta mappings related to the pretreatment robot-assisted movement and the posttreatment actively performed one. Finally, the authors acknowledge that the effect of the robotic intervention on the motor cortex was not quantified, due also to the low number of patients (one). Anyway, these data could be very important from the physiological point of view and could be the starting point for future studies on stroke rehabilitation. In any case, to overcome this limitation of the work, a study with pre- and posttreatment EEG evaluation will be performed on a group of stroke patients in the near future.

In conclusion, it was demonstrated that robotic full assistance does not inhibit brain activity but, conversely, it seems to promote cortical organization. Consistently with the literature, the presented data suggest that a procedure based on the use of EEG recordings and robot-assistance might be promising for assessing patients' potentialities for recovering. Further studies will be done to verify whether the approach may be used to set robot-assisted movements and assistive parameters or rather to customize the robotic treatment on the patients' picture.

## Supplementary Material

Videos 1 and 2 show the Hand-to-Mouth and the Reaching robot-assisted movements, respectively. To better define the patient's pathological picture before treatment videos 3 and 4 are provided. Functional gains at 12 months from the beginning of the treatment are shown in videos 5 and 6. The provided videos are hereafter summarized.
Video 1: Hand to Mouth robot-assisted movement.
Video 2: Reaching robot-assisted movement.
Video 3: Pretreatment active voluntary Hand to Mouth Movement.
Video 4: Pretreatment active voluntary Reaching Movement.
Video 5: Posttreatment (T3) active voluntary Hand to Mouth Movement.
Video 6: Posttreatment (T3) active voluntary Reaching Movement.

## Figures and Tables

**Table 1:** *α*-ERD, *β*-ERD, and *β*-ERS parameters on contralateral central electrode.

	Healthy subjects					
	*α*-ERD (%)	*α*-ERD (s)	*β*-ERD (%)	*β*-ERD (s)	*β*-ERS (%)	*β*-ERS (s)
Task A	−62.7 ± 21.4	1.5 ± 1.1	−63.0 ± 16.7	1.2 ± 1.0	77.5 ± 38.4	3.7 ± 1.1
Task B	−64.1 ± 28.3	1.2 ± 0.6	−66.4 ± 15.8	1.1 ± 0.5	81.2 ± 38.6	3.3 ± 0.9
Task C	−64.9 ± 20.0	2.3 ± 1.2	−65.1 ± 11.9	2.4 ± 1.2	74.7 ± 31.2	5.5 ± 0.8

**Table 2:** Patient's scores in the Fugl-Meyer Assessment and in the Manual Muscle Test.

Scale	(max. score)	T0	T1	T2	T3
FMA (0–66)					
Section A (shoulder and elbow)	(36)	17	23	30	35
Section B (wrist)	(10)	4	5	7	7
Section C (hand)	(14)	4	6	9	12
Section D (coordination/velocity)	(6)	4	5	5	5

Total score	(66)	**29**	**39**	**51**	**59**

MMT Scale (0–15)					
Shoulder abduction	(5)	4	4	5	5
Elbow extension	(5)	4	4	5	5
Fingers extension	(5)	1	1	1	3

Total score	(15)	**9**	**9**	**11**	**13**

FMA: Fugl-Meyer Assessment, MMT: Manual Muscle Test, T0: at the baseline, T1: after one month of treatment, T2: at the end of the treatment, and T3: at one-year follow-up.
