# Supplementary figures and images for: Predicting Functional Recovery in Chronic Stroke Rehabilitation Using Event-Related Desynchronization-Synchronization during Robot-Assisted Movement
Source: Biomed Res Int. 2016 Jan 17;2016:7051340. doi: 10.1155/2016/7051340 (PMC4739000; doi:10.1155/2016/7051340)

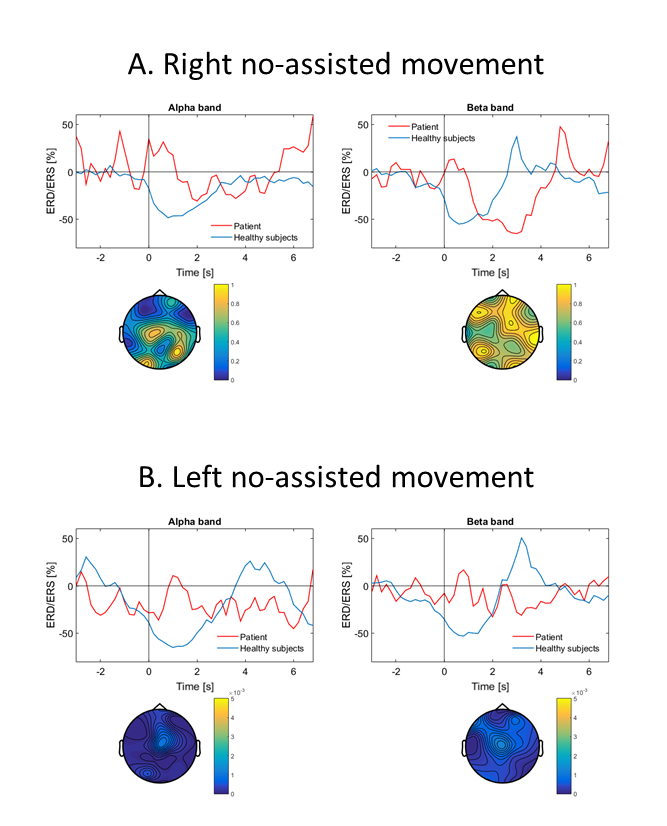

Supplement: Supplementary file 1 — Videos 1 and 2 show the Hand-to-Mouth and the Reaching robot-assisted movements, respectively. To better define the patient's pathological picture before treatment videos 3 and 4 are provided. Functional gains at 12 months from the beginning of the treatment are shown in videos 5 and 6. The provided videos are hereafter summarized. Video 1: Hand to Mouth robot-assisted movement. Video 2: Reaching robot-assisted movement. Video 3: Pretreatment active voluntary Hand to Mouth Movement. Video 4: Pretreatment active voluntary Reaching Movement. Video 5: Posttreatment (T3) active voluntary Hand to Mouth Movement. Video 6: Posttreatment (T3) active voluntary Reaching Movement. [file 7051340.f1.zip › No-Assisted Movement.png]
